# Book Reviews

**Published:** 1893-04

**Authors:** 


### Book Reviews.

A MANUAL OF MEDICAL JURISPRUDENCE, with Special Reference to Diseases and Injuries of the Nervous System. By ALLAN MCLANE HAMILTON, M. D., one of the Consulting Physicians to the Insane Asylums of New York City, etc., etc., with illustrations. New York: E. B. Treat, 5 Cooper Union. Chicago: 199 Clark street. 1890. Pp. 390. Price, $2.75.

As an authority on diseases of the nervous system and medical jurisprudence, Dr. Hamilton is conceded to be at the head, and hence his views on medico-legal topics must carry weight and conviction. In questions bearing on medico-legal cases, some authority must be followed, especially when the plea of insanity is entered by the defendant. Perhaps no branch of medicine calls forth more judgment and precision than those in which a human life is at stake, and hence the need of every physician to be more or less informed on medical jurisprudence. For such a treatise we can cheerfully recommend Allan McLane Hamilton's. The author divides his book into eight chapters, treating of Insanity, Insanity in its Medico-Legal Relations, Hysteroid Conditions and Feigned Diseases, Epilepsy, Alcoholism, Suicide, Cranial Injuries, and Spinal Injuries. Under Insanity the writer treats of questions bearing on the characteristics of the insane, their cunning, insane inspirations, relations of criminal acts to sleep, somnambulism and epilepsy, hereditary influence, etc. Under Chapter II. is reviewed briefly the Guiteau case; he also discusses the border-land of insanity, duties of the expert, testamentary capacity, and quotes in full the wills of certain individuals, made while they were suffering from insane ideas, etc., etc. We expected the author would give some extended information on the responsibility of the hysterical patient, but remarks only that it is far from easy and then quotes Du Saulle. This is one of the most important medico-legal topics and should be carefully studied. The responsibility of the epileptic is about settled, and the courts recognize that a criminal act during an attack of epilepsy is excusable. The author further ignores the question of responsibility of the hypnotized individual—in fact, no mention is made of hypnotism in its relation to crime. On the subject of spinal and cerebral concussion, with following suits for damages, the author takes middle ground, neither calling these cases all frauds, nor permitting simulation to be recognized as disease. To speak of all the interesting questions that the author discusses would be to reproduce the whole work. These questions are so interesting that it is difficult to lay the book aside before it has been carefully read.

The typography is similar to the other numbers of Treat's medical classics, combining beauty, strength and durability. W. C. K.

---

ALCOHOLISM AND ITS TREATMENT. By J. E. USHER, M. D., Fellow of the Royal Geographical Society of London, formerly Surgeon Superintendent and Medical Officer of Health to the Queensland Government. 12mo, pp. 151, cloth. New York: G. P. Putnam's Sons. London: Bailbére, Tindall & Cox. 1892.

This little book is written from the standpoint of one who recognizes that alcoholism is in some people a disease, that in others it induces disease, and that in others it is a vicious habit. In establishing alcoholism as a disease, it is not intended that any forms other than those of a chronic nature, or when a known heredity exists, or in which fixed mental change (insanity) is present, can be allowed in palliation of any offense.

In three chapters he discusses pathological changes in alcoholism, the nature of the inherited, the acquired, and the infantile forms.

The next four chapters consider insanity alcoholism, alcoholic trance and crime, cerebral automatism or trance, alcoholism and its legal relations.

These chapters are exceedingly well written and full of sound and valuable information. The chapter which considers the legal status of drunkards is a resumé of the conditions in England and New York. He concludes that it will be observed that the law has not yet judicially recognized inebriety as a disease.

The remainder of the book is given to the treatment of alcoholism. In this is included the following prescriptions; the first is the alleged bichloride of gold remedy sold at Dwight, which, on being analyzed, gives the following result:

℞—Ammon. muriat.... ................gr. i.
Aloin..............................gr. ii.
Tinct. cinch. co. ....................℥iii.
Aq.......................... .....℥i.

M. S. Teaspoonful six times daily.

The hypodermic injection used at Dwight shows an analysis of—

℞—Strych. sulph.........................gr. ss.

Atropia. .... .......................gr. ¼.

Acid borac. .........................gr. xv.

Aq.............................℥ iv.

J. W. P.

---

THE READY-REFERENCE HAND-BOOK OF DISEASES OF THE SKIN. By GEORGE THOMAS JACKSON, M. D. (Col.), Chief of Clinic and Instructor in Dermatology, College of Physicians, New York; Professor of Dermatology in Woman's Medical College of the New York Infirmary; Consulting Dermatologist to the Presbyterian Hospital; Visiting Dermatologist to the Randall's Island Hospitals; Member of the American Dermatological Society; Fellow of the New York Academy of Medicine, etc.

The object of this Ready-Reference Hand-book, as the author sets forth in his preface, is "to present the art of dermatology as it now exists." This has been well done, the compendium being up to date, full of scientific accuracy, and conveniently arranged for the practical adaptations to the wants of the dermatological student.

The diseases follow in alphabetical order, their salient features clearly described, and their diagnosis and treatment fully considered. The concluding portion of the work consists of an appendix containing additional formulæ, wherein the grains, drachms, and ounces are translated into their precise equivalents in grammes.

The style and diction are admirable, and we cannot too highly commend this compact treatise as a faithful presentation of the present state of our knowledge of dermatology. E. W.

---

The Anatomy of the Peritoneum. By Franklin Dexter, M. D., Assistant Demonstrator of Anatomy, College of Physicians and Surgeons (Columbia University), New York. With thirty-eight illustrations ; 12mo, pp. 86. New York : D. Appleton & Co. 1892.

Both teacher and student will unite in thanking the author for giving them such a clear and comprehensive description of the peritoneum. The subject is one with which every teacher of anatomy has experienced difficulty when trying to explain it to the student, and this little book cannot fail to help him greatly in his task.

As the author frankly states, while he cannot at all times explain why anatomical conditions occur, in some cases, we can understand how such conditions are produced, and such knowledge is better than that which is purely a matter of memory. The keynote of this book is the study of the development of the abdominal organs, and no one, after reading what the author has to say along these lines, can fail to appreciate the value of the method.

The author begins with the peritoneum in its most elementary form, and, step by step, with text and accompanying illustration, he traces its development from the time when the alimentary canal is a simple tube up to the more complex arrangement of the human adult. The book cannot easily be reviewed, inasmuch as much of its clearness depends upon the plates, and these must, of course, be seen. The plates are exceedingly distinct and simple, and are the great feature of the book. The text makes an admirable supplement and aids the interpretation of the plates.

There are few things to criticize. One or two typographical errors and the retaining of the diphthong in the word peritoneum are about the only blemishes to be found.

The little volume is very dainty in its make-up, and suggests that the time is coming when a medical book need not necessarily look like one, and that hard, dry science may have as attractive clothing as poetry or art. J. P.

---

A Manual of Clinical Ophthalmology, by Howard F. Hansell, M. D., Lecturer on Ophthalmology in the Jefferson Medical College; Chief Clinical Assistant in the Eye Department, Jefferson Medical College Hospital; Member of American Ophthalmological Society; Fellow of the College of Physicians, Philadelphia, etc., and James H. Bell, lately Demonstrator of Anatomy in Jefferson Medical College; Member Ophthalmological Staff, Jefferson Medical College Hospital; Ophthalmic Surgeon to Southwestern Hospital and Dispensary, etc. With 120 illustrations. Pp. 231. Philadelphia: P. Blakiston, Son & Co. 1892. Price, $1.75.

This is a manual designed to give "a brief review of the anatomy, physiology, refraction, and common diseases of the eye." The authors have written it for "the undergraduate and general practitioner of medicine." The subjects are presented in very clear and concise style, and so far as treated, are brought fully up to date. The scope of the work forbids that detail which is often desirable, and excludes some topics that are of very little practical importance to the student or general practitioner. Very few errors are noticed, the most apparent one being the misnaming of Priestley Smith's perimeter.

The authors have done their work well, and have produced a book that can be sincerely recommended to the medical student who needs a concise and compendious presentation of the essentials of ophthalmology. A. A. H.

---

Manual of Skin Diseases with Special Reference to Diagnosis and Treatment, for Students and General Practitioners. By W. A. Hardaway, M. D., Professor of Skin Diseases in the Missouri Medical College, and in the St. Louis Post-Graduate School of Medicine; Dermatologist to the Augusta Free Hospital for Children; Consulting Dermatologist to the City and Female Hospitals; ex-President of the American Dermatological Association.

This little manual is divided into three parts, the first portion being devoted to a general introduction on Symptomatology, Etiology, Diagnosis, and Treatment, which is brief, practically complete, and clearly stated.

The second part considers the clinical characters of the various skin affections in a most satisfactory manner, special stress being placed upon diagnosis and treatment.

It concludes with an appendix of special formulæ selected by the author, and a diet table. E. W.

FORMULAIRE DES MEDICAMENTS NOUVEAU ET DES MEDICATIONS NOUVELLES POUR 1893, par H. BOCQUILLON-LIMOUSIN, Pharmacien de 1re Classe, avec une introduction par H. Huchard, médecin de l'hôpital Bichat. 1 vol. in-18 de 320 pp., cart. 3 fr. Libraririe J.-B. Billière et fils 19, rue Hautefeuille (près du boulevard Saint-Germain), à Paris.

A large number of new remedies have of late been introduced into therapeutics. The indications for their use, their origin, and mode of employment are scattered through the medical literature of the world. The object of this little work has been to collect and condense these reports and to present them to the profession in such a way that at a glance he can find what would otherwise consume valuable time.

Among the 500 important articles which it contains may be mentioned antipyrine, exalgine, ichthyol, menthol, naphthaline, salol, salophen, spermine, sulfonal, analgene, asaprol, strontium, etc., enough to show that the very latest additions to our therapeutic knowledge have been incorporated.

The low price (sixty cents) makes it accessible to the mass of practising physicians, who will find it many times worth its intrinsic value. W. C. K.

---

PUBLIC LEDGER ALMANAC, 1893.—GEO. W. CHILDS, Publisher, Chestnut street, Philadelphia.

It is our pleasure to again notice this valuable almanac. The one for last year seemed very near perfection, but this, if anything, is an improvement on the last one. It contains a record of the important events of this year, also a list of the members of Congress, and of the Diplomatic corps, and many other interesting items. This is the twenty-fourth year of its publication, and as the *Ledger* Building was burned on December 5, 1892, it was published under many difficulties.

---

### BOOKS RECEIVED.

Proceedings of the Philadelphia County Medical Society. Volume XIII. Session of 1892. Louis H. Adler, Jr., M. D., Editor. Philadelphia. Printed for the Society. 1893.

Human Anatomy. A Complete Systematic Treatise by Various Authors, including a Special Section on Surgical and Topographical Anatomy. Edited by Henry Morris, M. A., and B. M., Lond., Surgeon to, and Lecturer on Surgery, formerly Lecturer on Anatomy at the Middlesex Hospital, late Examiner in Anatomy in the University of

Burham, and for the Royal College of Physicians on the Conjoint Board. Illustrated by 791 woodcuts, 214 of which are printed in colors from drawings made expressly for this work by special artists. Large octavo, pp. xxxii.—1286. Price, $7.50. Philadelphia: P. Blakiston, Son & Co., 1012 Walnut street. 1893.

A Year-Book of Treatment for 1893, a critical review for practitioners of medicine and surgery. Contributors: Barclay J. Baron, M. B.; Stanley Boyd, B. S., F. R. C. S.; J. Mitchell Bruce, M. D.; Alfred Cooper, F. R. C. S.; W. H. Corfield, M. D.; George P. Field, M. R. C. S.; Archibald E. Garrod, M. D.; Reginald Harrison, F. R. C. S.; G. Ernest Herman, M. B.; J. Ernest Lane, F. R. C. S.; Robert Maguire, M. D.; Malcolm Morris, F. R. C. S. E.; Edmund Owen, F. R. C. S.; Sidney Phillips, M. D.; Henry Power, F. R. C. S.; Charles Henry Ralfe, M. D.; E. S. Reynolds, M. D.; E. Markham Skerritt, M. D.; Walter G. Smith, M. D.; W. J. Walsham, F. R. C. S. Small octavo pp.—496. Lea Brothers & Co., Philadelphia. 1893.

The Students' Quiz Series. Edited by Bern B. Gallaudet, M. D., Demonstrator of Anatomy and Clinical Lecturer on Surgery, College of Physicians and Surgeons, New York. Volume VIII. Diseases of the Skin, by Charles C. Ransom, M. D., Assistant Dermatologist, Vanderbilt Clinic, New York. Pocket size, 12mo, 192 pages, twenty-eight illustrations. Limp cloth, $1.00. Philadelphia: Lea Brothers & Co. 1893.

Disease in Children, a manual for students and practitioners. By James Carmichael, M. D., F. R. C. P., Ed. Physical Royal Hospital for Sick Children; University Lecturer on Disease in Children, Edinburgh. Illustrated with thirty-one charts, pp. xvi.—591. Small octavo. New York: D. Appleton & Co. 1893.

International Clinics. A Quarterly of Clinical Lectures on Medicine, Neurology, Pediatrics, Surgery, Genito-Urinary Surgery, Gynecology, Ophthalmology, Laryngology, Otology, and Dermatology, by professors and lecturers in the leading medical colleges of the United States, Great Britain, and Canada. Edited by John M. Keating, M. D., LL. D., Colorado Springs, Col., Fellow of the College of Physicians, Philadelphia; formerly Consulting Physician for Diseases of Women to St. Agnes' Hospital; Gynecologist to St. Joseph's Hospital; Visiting Obstetrician to the Philadelphia Hospital, and Lecturer on the Diseases of Women and Children, Philadelphia; Editor Cyclopedia of the Diseases of Children. Judson Daland, M. D., Philadelphia, Instructor in Clinical Medicine and Lecturer on Physical Diagnosis and Symptomatology in the University of Pennsylvania; Assistant Visiting Physician to the University Hospital; one of the Examiners of the Insane to the Philadelphia Hospital; Visiting Physician to St. Clement's Hospital, Philadelphia. J. Mitchell Bruce, M. D., F. R. C. P., London, Eng., Physician and Lecturer on Therapeutics at the Charing Cross Hospital.

David W. Finlay, M. D., F. R. C. P., Aberdeen, Scotland, Professor of Practice of Medicine in the University of Aberdeen, Physician to and Lecturer on Clinical Medicine in the Aberdeen Royal Infirmary; Consulting Physician to the Royal Hospital for Diseases of the Chest, London. Volume IV. Second series. 1893. Octavo, pp. xxiii.—387. Philadelphia: J. B. Lippincott Co. 1893.

